# Supplementary material for: The Applicability of a 2-Transcript Signature to Identify Bacterial Infections in Children with Febrile Neutropenia
Source: Children (Basel). 2023 May 29;10(6):966. doi: 10.3390/children10060966 (PMC10297134; doi:10.3390/children10060966)
Supplement: Supplementary file 1 [file children-10-00966-s001.zip › children-2319057-supplementary.pdf]

Supplemental Table S1

Table S1. Clinical characteristics of the re-classified patients. Patients A through I were previously classified as unknown pathogen; J – N previously classified as viral. Determination of WBC count was done the same day as collection of RNA samples.

	**Probable Viral Infection**			**Probable Bacterial Infection**										
**Age (years)**	1.6	0.6	6.5	0.5	7.4	5.1	15.8	9.4	16.0	16.0	9.3	9.9	16.1	15.9
**Gender**	F	M	F	F	F	F	M	F	M	F	F	F	M	F
**Diagnosis**	S	S	LL	LL	CNS	S	S	CNS	S	LL	S	S	S	S
**Peak CRP (mg/L)**	16	5	4	206	124	115	130	412	103	215	120	214	145	240
**WBC count (10^9/L)**	5.6	1.6	1.6	2.0	0.7	0.7	0.4	0.8	14.6	0.3	0.4	0.4	2.2	1.3
**ANC (10^9/L)**	0.6	0.1	0.2	<0.1	0.2	0.2	<0.1	0.5	<0.1	<0.1	<0.1	<0.1	0.2	<0.1
**Days with neutropenia**	7	13	12	>30	9	6	3	14	1	4	6	3	2	6
**Peak Temp (°C)**	39.2	38.1	38.9	39.0	39.0	38.4	39.3	39.3	38.8	38.9	38.1	39.2	39.2	40.0
**Days with fever**	3	2	2	16	1	4	2	1	2	3	1	3	2	4
**Days hospitalized**	2	5	3	>30	5	4	5	5	5	7	6	9	5	7
**Days with antibiotics**	0	5	4	30	7	11	5	9	5	7	6	8	5	9
**Respiratory symptoms**	Yes	No	Yes	Yes	Yes	Yes	Yes	Yes	Yes	Yes	Yes	No	Yes	No
**Gastrointestinal symptoms**	No	No	No	Yes	No	No	No	No	No	Yes	No	No	No	No
**Local symptoms**	None	None	None	None	None	None	None	None	None	None	None	None	None	None
**Microbial findings**	None	None	None	None	None	None	None	None	None	Infl A	Rhino/ Entero*	Rhino	Parainfl 3	Rhino/ Entero*

Abbreviations: LL, Leukemia and lymphomas; S, solid tumour; CNS, central nervous system malignancy.
*Known cross reaction between rhinovirus and enterovirus through PCR analysis.

Supplemental Table S2. Top 20 significant* up- and downregulated genes

PVI vs C				PBI vs C				PBI vs PVI			
Probe	P-value	FDR	Log2 Fold Change	Probe	P-value	FDR	Log2 Fold Change	Probe	P-value	FDR	Log2 Fold Change
MIXL1	1,97E-06	1,64E-04	5,138326645	IGLL1	1,19E-08	1,73E-04	5,758036137	PRTN3	6,57E-11	8,69E-07	5,297813
UCHL1	9,25E-05	3,20E-03	4,623913765	MYCN	1,85E-05	2,38E-02	4,386828899	CTSG	3,76E-11	8,69E-07	4,833078
OTOF	6,97E-07	7,79E-05	4,060622692	CTSG	3,58E-07	1,58E-03	4,103831291	GSTM1	2,43E-08	9,20E-05	4,740152
HRASLS2	1,11E-04	3,68E-03	3,74836421	CYP19A1	1,05E-05	2,12E-02	3,958764553	CAPN13	1,53E-05	4,64E-03	4,62509
PBK	1,80E-08	5,29E-06	3,544628382	NPR3	1,15E-05	2,16E-02	3,94164753	MMP2	1,07E-05	4,14E-03	4,441769
GPRC5D	7,18E-05	2,68E-03	3,524526358	DEFA4	4,41E-05	3,90E-02	3,842700958	ELANE	1,49E-08	6,57E-05	4,39225
RP11-161H23.9	5,40E-07	6,35E-05	3,517868757	AP3B2	1,59E-05	2,20E-02	3,796952486	NNMT	2,77E-04	2,14E-02	4,3543
SPC25	7,18E-07	7,95E-05	3,516967535	ELANE	1,29E-05	2,16E-02	3,715790272	IL1R2	2,37E-09	2,09E-05	4,281482
TNFRSF17	9,72E-04	1,81E-02	3,504294634	MPO	5,35E-07	2,02E-03	3,631379366	RAB17	1,39E-04	1,56E-02	4,276853
IGLL1	1,67E-03	2,68E-02	3,454075336	DNTT	5,79E-05	4,02E-02	3,5413661	AC000032.2	1,20E-04	1,44E-02	4,230737
MND1	5,36E-06	3,66E-04	3,37774229	ANO7	6,73E-05	4,44E-02	3,533829689	LYVE1	1,75E-06	1,71E-03	4,120498
IGLL5	1,49E-03	2,47E-02	3,319755793	ZDHHC19	7,13E-05	4,48E-02	3,408807039	CALN1	2,15E-04	1,99E-02	3,958011
MZB1	5,91E-06	3,95E-04	3,302513599	CD177	5,29E-05	3,93E-02	3,350853443	DEFA4	1,65E-06	1,71E-03	3,944625
BHLHA15	8,72E-04	1,68E-02	3,247926712	CPXM1	1,31E-08	1,73E-04	3,230443239	AZU1	4,50E-07	8,50E-04	3,78856
TNFRSF13B	9,08E-04	1,73E-02	3,208951473	CD34	2,95E-06	7,28E-03	3,106764793	CTB-50L17.14	1,81E-07	4,78E-04	3,739308
FAM154B	5,38E-04	1,19E-02	3,181321621	CYTL1	5,36E-05	3,93E-02	2,58163166	RP11-314C9.1	2,76E-04	2,14E-02	3,723403
AC096579.13	1,44E-03	2,41E-02	3,145660877	HSD3B7	2,24E-05	2,57E-02	2,163443327	CYP19A1	1,40E-05	4,63E-03	3,702624
AC007386.4	1,32E-03	2,25E-02	3,118636131	CDC25A	5,63E-05	4,01E-02	2,10907793	MPO	3,43E-09	2,27E-05	3,646697
RRM2	1,51E-09	9,35E-07	3,067816257	ATP2C2	7,30E-05	4,48E-02	2,082764149	MIR371B	1,10E-04	1,36E-02	3,590461
NDUFA4L2	9,24E-04	1,76E-02	3,039817095	GINS2	3,04E-06	7,28E-03	2,060665131	AP3B2	5,43E-06	2,71E-03	3,543175
KIAA1045	1,81E-03	2,83E-02	-4,386102676	DDX58	5,14E-08	3,39E-04	-1,016492248	C6orf52	7,86E-05	1,17E-02	-1,469691634

TSPEAR	2,32E-08	6,39E-06	-4,402029037	EIF2AK2	3,87E-06	8,51E-03	-1,146949172	CFB	7,21E-04	4,01E-02	-1,663973212
AC007950.1	1,05E-03	1,92E-02	-4,430828094	ZBP1	2,24E-05	2,57E-02	-1,148426533	BMS1P20	8,00E-05	1,18E-02	-1,699842811
CTD-2530H12.2	1,97E-04	5,71E-03	-4,463878632	HERC5	2,68E-06	7,28E-03	-1,392508268	MYOM2	2,36E-04	2,02E-02	-1,788220644
FAM169B	3,37E-03	4,45E-02	-4,464846134	CMPK2	7,02E-05	4,48E-02	-1,500822663	MX1	3,04E-05	6,59E-03	-1,814431548
AC008074.3	7,56E-04	1,53E-02	-4,467960835	RSAD2	1,52E-05	2,20E-02	-1,898011088	CH507-42P11.8	1,25E-04	1,49E-02	-1,816319704
CXCL6	3,09E-04	8,00E-03	-4,549657345	IFIT1	2,62E-05	2,88E-02	-2,196577787	AP001610.5	1,36E-05	4,55E-03	-1,877956271
RP11-556I13.2	3,32E-09	1,67E-06	-4,601095676	IFIT3	4,73E-05	3,90E-02	-2,220742702	CADM1	2,41E-04	2,03E-02	-1,895445108
LYVE1	1,33E-09	8,43E-07	-4,684877872	IFIT2	2,21E-07	1,17E-03	-2,490629196	ISG15	1,70E-05	5,04E-03	-1,913071513
FABP6	1,46E-04	4,57E-03	-4,695086956	CCDC64B	2,45E-08	2,15E-04	-3,056697607	OAS3	5,87E-06	2,87E-03	-1,987203836
RP11-213H15.1	3,31E-06	2,50E-04	-4,814025402					PLS3	2,77E-04	2,14E-02	-2,070141792
GRAMD1C	2,95E-09	1,59E-06	-4,94207716					SAMD4A	3,33E-06	2,44E-03	-2,140824795
RP11-701P16.2	3,32E-04	8,37E-03	-4,951215744					LAMP3	2,22E-05	5,76E-03	-2,173040152
RP11-255E6.6	1,01E-03	1,86E-02	-4,967195988					AXL	1,92E-04	1,88E-02	-2,276320934
RP11-321E2.4	2,41E-04	6,57E-03	-4,970205784					RSAD2	4,95E-06	2,68E-03	-2,43867445
RP11-875O11.2	6,40E-05	2,46E-03	-4,978557587					USP18	5,63E-07	9,93E-04	-2,558294058
RP11-7F17.3	6,39E-07	7,23E-05	-5,47255373					AP001626.1	5,41E-04	3,35E-02	-3,112215996
OTX1	4,49E-07	5,73E-05	-5,527425289					IFI27	2,70E-04	2,14E-02	-3,415747166
DAAM2	6,91E-04	1,43E-02	-6,166125298					OTOF	3,14E-07	6,93E-04	-4,034413815
RP11-561P12.5	8,99E-07	9,10E-05	-6,727097988					RP11-7I15.3	1,71E-06	1,71E-03	-4,372183323
**FOU vs C**				**FOU vs PVI**				**FOU vs PBI**			
**Probe**	**P-value**	**FDR**	**Log2 Fold Change**	**Probe**	**P-value**	**FDR**	**Log2 Fold Change**	**Probe**	**P-value**	**FDR**	**Log2 Fold Change**
IGLL1	4,70E-07	0,002426	5,744669	EDIL3	8,72E-04	0,049946	4,502246	RP11-293A21.1	6,76E-06	0,035666	4,114618
VAT1L	1,76E-04	0,031081	5,358256	CTSG	5,48E-09	1,42E-04	4,191145	TMEM158	5,46E-07	0,0036	3,2096
MYCN	1,35E-05	0,009934	4,522799	CD207	1,19E-04	0,021285	3,906018	SYCP3	1,01E-05	0,044438	2,57213
AJ006998.2	3,91E-04	0,046442	4,470375	ELANE	5,07E-06	0,00813	3,880461	GFRA2	8,59E-09	2,27E-04	2,004329
LINC00607	2,53E-06	0,007259	4,301623	PRTN3	1,48E-05	0,013356	3,779587	SAMD4A	4,55E-07	0,0036	1,764694

NPR3	1,86E-07	0,002397	4,14112	CALN1	8,25E-04	0,048773	3,672753	ACCS	1,04E-07	0,001367	1,396393
MAFA	1,01E-04	0,024263	4,128905	TM4SF1	6,77E-06	0,009221	3,646864				
ANO7	4,28E-04	0,047573	4,122278	DEFA4	1,24E-04	0,021427	3,480872				
SHANK3	2,51E-06	0,007259	3,940799	SLPI	4,87E-06	0,00813	3,462449				
HRASLS2	2,04E-04	0,034682	3,814175	ZNF521	1,37E-05	0,013091	3,455169				
RP11-161H23.9	3,84E-04	0,04616	3,599853	STOX2	5,02E-04	0,04009	3,447867				
NRARP	3,21E-05	0,01341	3,56766	AZU1	1,88E-06	0,007068	3,427548				
CD34	6,43E-09	1,66E-04	3,566763	CEACAM6	7,17E-04	0,04562	3,389188				
CTSG	1,46E-05	0,009934	3,483392	MPO	8,54E-07	0,005529	3,358277				
CPXM1	4,56E-06	0,007736	3,365699	HSPA4L	4,77E-06	0,00813	3,274673				
MPO	3,68E-05	0,014632	3,364521	RP11-314C9.1	4,14E-04	0,037315	3,245683				
AC023590.1	1,36E-04	0,027887	3,298299	MMP8	5,76E-04	0,042543	3,143515				
THEM5	4,51E-06	0,007736	3,105394	HTRA3	4,77E-05	0,015889	3,09043				
SPC25	8,96E-05	0,022247	3,090211	CHI3L1	3,29E-05	0,015496	3,085031				
FAM132B	8,89E-05	0,022247	3,08235	LTF	6,43E-04	0,044033	3,046068				
ERN1	2,62E-04	0,038688	-1,00417	TRBV18	4,02E-04	0,037315	-1,05544				
CARD8	2,71E-04	0,039301	-1,00419	TRAV29DV5	8,66E-04	0,049698	-1,08548				
CXCR4	1,07E-04	0,024863	-1,00941	TRAV20	7,01E-04	0,045131	-1,11158				
TRANK1	6,89E-06	0,007736	-1,01768	TRBV5-1	3,70E-04	0,035506	-1,13537				
AHCTF1	3,99E-04	0,046442	-1,05061	ADAMTS4	5,34E-04	0,041089	-1,17677				
RP13-20L14.10	8,77E-05	0,022247	-1,05482	HERC6	8,46E-04	0,04945	-1,22344				
LINC00987	4,46E-05	0,016032	-1,0561	AGMAT	9,82E-05	0,020293	-1,2238				
LITAF	1,44E-04	0,027887	-1,06794	TRAV2	6,86E-04	0,044646	-1,2423				
ABCB1	3,97E-04	0,046442	-1,06933	TRAV38-2DV8	8,98E-05	0,019218	-1,2453				
PRR33	2,20E-04	0,035122	-1,10142	TRBV7-3	4,69E-04	0,039986	-1,25605				
NLGN3	3,05E-04	0,041547	-1,10388	TRAV26-1	9,91E-05	0,020293	-1,26969				
NOTCH1	1,77E-04	0,031081	-1,10544	TRAV8-6	2,59E-04	0,029942	-1,28527				

**PARP8**	**1,16E-04**	**0,026411**	**-1,10898**	**TMPRSS3**	**1,37E-04**	**0,022609**	**-1,2943**				
**ABCG1**	**9,36E-06**	**0,009292**	**-1,11521**	**LINC00565**	**1,00E-04**	**0,020293**	**-1,36752**				
**TMCC1**	**1,84E-04**	**0,03203**	**-1,16234**	**TRAJ20**	**3,26E-04**	**0,033508**	**-1,38584**				
**ABHD3**	**3,34E-04**	**0,043285**	**-1,16272**	**TRBV10-3**	**4,19E-05**	**0,015889**	**-1,3959**				
**RP11-327P2.5**	**4,99E-04**	**0,049584**	**-1,18444**	**TRAV25**	**5,46E-05**	**0,015889**	**-1,40021**				
**AC116366.6**	**5,02E-06**	**0,007736**	**-1,1888**	**TRAJ49**	**7,47E-05**	**0,018175**	**-1,41402**				
**TNFSF14**	**4,46E-04**	**0,047657**	**-1,21442**	**TRAV22**	**6,11E-05**	**0,016285**	**-1,41851**				
**FAM65B**	**6,08E-05**	**0,019389**	**-1,22534**	**TRAV41**	**2,27E-05**	**0,015262**	**-1,42564**				

[*]Significant genes: false discovery rate (FDR) < 0.05 and a fold change (FC) difference of at least 2.

PVI=probable viral infections, PBI=probable bacterial infections, C=controls, red=up-regulated genes, blue=down regulated genes
